# New Optic Nerve Sonography Quality Criteria in the Diagnostic Evaluation of Traumatic Brain Injury

**DOI:** 10.1155/2018/3589762

**Published:** 2018-04-30

**Authors:** Ibrahim Soliman, Garrett G. R. J. Johnson, Lawrence M. Gillman, Frederick A. Zeiler, Fahad Faqihi, Waleed Tharwat Aletreby, Abdullah Balhamar, Nasir Nasim Mahmood, Shahzad Ahmad Mumtaz, Abdulrahman Alharthy, Christos Lazaridis, Dimitrios Karakitsos

**Affiliations:** ^1^Neurocritical Care Unit, Critical Care Department, King Saud Medical City, Riyadh, Saudi Arabia; ^2^Undergraduate Medical Education, Rady Faculty of Health Sciences, University of Manitoba, Winnipeg, MB, Canada; ^3^Department of Surgery, Rady Faculty of Health Science, University of Manitoba, Winnipeg, MB, Canada; ^4^Clinician Investigator Program, Rady Faculty of Health Sciences, University of Manitoba, Winnipeg, MB, Canada; ^5^Division of Anesthesia, Addenbrooke's Hospital, University of Cambridge, Cambridge, UK; ^6^Division of Neurocritical Care, Departments of Neurology and Neurosurgery, Baylor College of Medicine, Houston, TX, USA; ^7^Department of Critical Care, Keck Medical School, USC, Los Angeles, CA, USA

## Abstract

**Background:**

New sonographic quality criteria to optimize optic nerve sheath diameter (ONSD) measurements were suggested. The latter were correlated to elevated intracranial pressure (ICP) in traumatic brain injury (TBI).

**Aim:**

We investigated whether ONSD measurements were correlated to simultaneous ICP measurements in severe TBI.

**Methods:**

Forty patients with severe TBI (Marshall Scale ≥II and GCS ≤8) participated in the study. All patients had an intraparenchymal ICP catheter inserted, while ONSD was measured bilaterally, upon admission and over the next 48 hours, based on the new sonographic criteria. A total of 400 ONSD measurements were performed, while mean ONSD values of both eyes were used in the analysis.

**Results:**

ONSD measurements were strongly correlated to ICP values (*r*=0.74, *p* < 0.0001). Receiver operator curve (ROC) analysis revealed that the ONSD cutoff value for predicting elevated ICP was 6.4 mm when using the mean of both eyes (AUC = 0.88, 95% CI = 0.80 to 0.95; sensitivity = 85.3%, specificity = 82.6%). Linear regression analysis nested models revealed that sex (*p*=0.006) and height (*p*=0.04) were significant predictors of ONSD values.

**Conclusion:**

When applying the new sonographic quality criteria, ONSD is strongly correlated to ICP in severe TBI. Whether to use such criteria to monitor ONSD as a proxy for ICP trend in TBI remains to be further explored.

## 1. Introduction

Traumatic brain injury (TBI) is frequently complicated by elevated intracranial pressure (ICP). Secondary brain injury due to elevated ICP and decreased perfusion pressure to the brain is an important cause of morbidity and mortality in those patients. In order to treat this complication, elevated ICP must be diagnosed quickly and accurately [1–3]. Direct monitoring of ICP through insertion of an intracranial monitor is considered the gold standard in the diagnosis of intracranial hypertension [4]. Due to the invasive nature of these procedures and associated risks, intracranial monitoring is usually not employed until after elevated ICP is already suspected based on clinical picture and noninvasive testing such as computed tomography [5]. Additionally, invasive monitoring may not always be possible due to coagulopathy, thrombocytopenia, or lack of relevant procedural expertise and tools [6].

**Figure 1:**
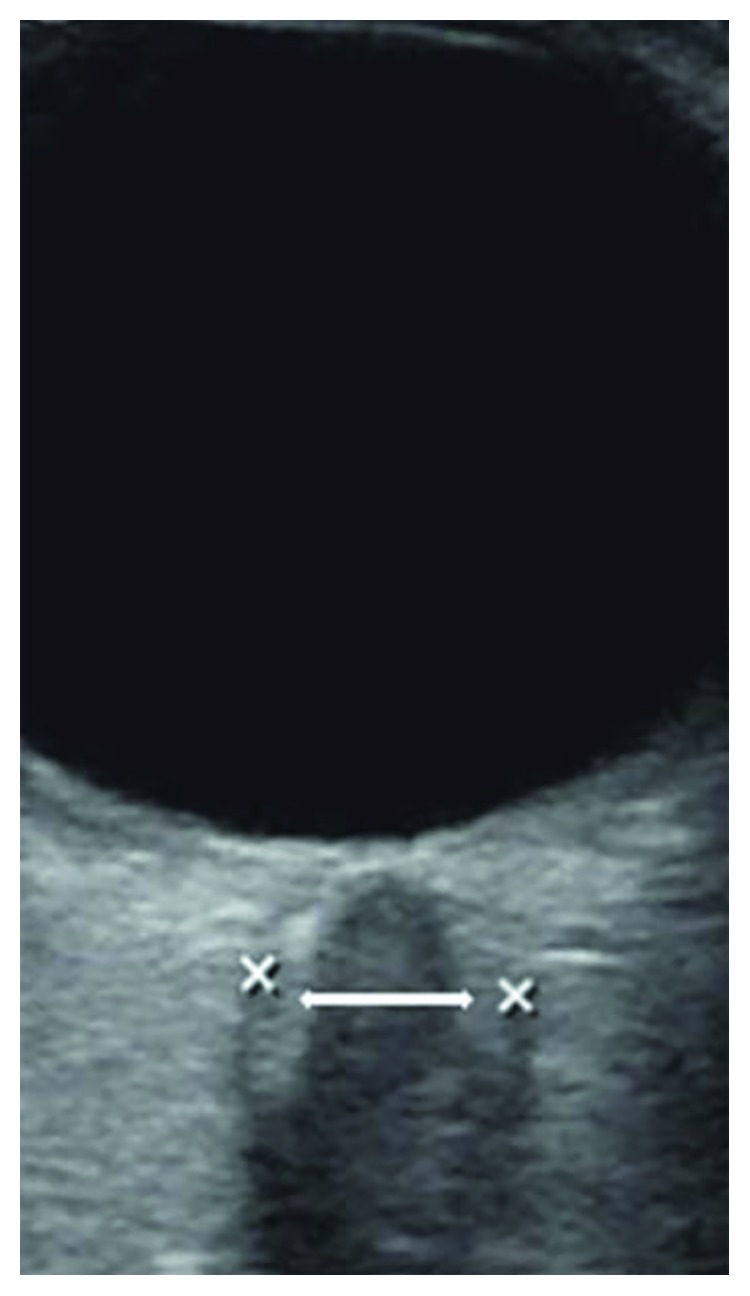
Employment of the “black stripe” method (white arrow) may underestimate the actual optic nerve sheath diameter (ONSD) measurement (*x*–*x*) based on the new sonographic quality criteria in patients with increased intracranial pressure.

Ultrasound assessment of optic nerve sheath diameter (ONSD) has been assessed as a promising tool to aid in the diagnosis of elevated ICP both in TBI and various nontraumatic brain-injured patients [7, 8]. The optic nerve sheath (ONS) is contiguous with the dura matter surrounding the brain and contains cerebrospinal fluid, which allows transmission of pressure from the cranium [9]. In previous studies, acute increases in ICP have correlated strongly with increases in ONSD [8, 10, 11]. However, there is still some disagreement about the recommended threshold value above which a certain ONSD should indicate a pathological increase in ICP. Recommendations for these cutoff values have varied from 5.0 to 5.9 mm with sensitivities and specificities ranging from 70 to 100% and 30 to 100%, respectively, depending on the study and the optimal cutoff value identified [7, 8, 10]. There is also some dispute about what the normal range of ONSD is in healthy individuals, and how age, height, weight, sex, and ethnicity affect this [12–16]. Part of this variance may be due to the lack of rigorous standardization for obtaining quality images of ONSD. Currently, the most popular method studied in the literature for evaluating ONSD is the “black stripe” method and involves identifying the ONS as a black line behind the globe and measuring its diameter 3 mm behind the papilla [11, 17–20]. Recently, new sonographic quality criteria for optimizing ONSD measurements in critical care settings were suggested as a way to standardize measurements across different sonographers and scans and to improve image quality (Table 1; Figure 1) [21]. However, the new quality criteria have not been prospectively evaluated in the literature.

In this study, we compared ONSD measured by the application of the new quality criteria to invasive ICP evaluated simultaneously by intraparenchymal catheter in intensive care unit (ICU) patients with severe TBI. Hence, we aimed on identifying an optimal ONSD threshold value corresponding to elevated ICP.

## 2. Patients and Methods

### 2.1. Patients

This prospective study was performed from January to September 2017 at the Neurocritical Care Unit (NCCU) of the polyvalent ICU department (King Saud Medical City, Riyadh, KSA). It is an ongoing registered study (ISRCTN 33349) performed by our group to explore further the role of ONSD sonographic monitoring in brain-injured patients. In this report, we included only adult patients (>18 years old) who were admitted to the ICU with severe TBI. Patients with orbital trauma and/or known optic nerve pathology were excluded from the study. Severity of brain injury was graded according to a combination of Glasgow Coma Scale (GCS) and brain computed tomography scan derived Marshall Scale (I-VI) as previously described [22]. Power sample analysis determined that 400 ONSD measurements would provide approximately 90% statistical power (alpha = 0.05, one-sided) to compare the former to invasive ICP values.

Upon hospital admission, all patients with severe TBI (GCS ≤8 and Marshal Scale ≥II) underwent clinical evaluation by a multidisciplinary team of experts including neurosurgeons and neurointensivists. Patients were transferred to a specialized 16-bed neurocritical care unit within the premises of the polyvalent 120-bed ICU whether or not a neurosurgical intervention was performed depending on the clinical case scenario. All patients were closely monitored and placed under brain protective strategy in the ICU. Briefly, in all patients mean arterial blood pressure (MAP) was continuously monitored using an invasive arterial catheter to exclude hypotension (systolic blood pressure <110 mmHg). Heart rate was monitored to exclude bradycardia (heart rate <60 beats/minute), and pulse oximetry was initiated to exclude hypoxemia (arterial oxygen saturation <95%). Upon ICU admission, all patients were sedated and mechanically ventilated (volume-controlled continuous mandatory ventilation mode), and arterial carbon dioxide tension was maintained at 33 to 35 mmHg throughout the study period. Sedation vacation was performed on daily regular intervals to evaluate patients' GCS and neurological status. Families' consent was obtained in all cases for participation in the study. The latter conformed to the principles outlined in the Declaration of Helsinki and was approved by the institutional ethics committee.

### 2.2. Methods and Data Collection

Sonographic examinations were conducted using Philips HD11XE (Philips Medical Systems; Bothell, WA, USA) equipped with a 10–20 MHz linear transducer. All patients were examined in the supine position as previously described [17–22], the exam takes less than a minute (images stored and analyzed later), and then the patient's head immediately repositioned to 30 degrees head up position as per TBI protocol. The ONSD was measured according to the new quality criteria as detailed elsewhere (Table 1) [21]. The ONSD was recorded for both eyes in all cases, while the mean value of all measurements which were electronically stored and reviewed by expert sonographers was used in the statistical analysis. Initial sonographic scans were performed 15–20 minutes after a CT scan to determine severity of brain injury and eligibility for the study. Both eyes were scanned for each patient, and the mean of both readings was correlated to simultaneous ICP recordings. ONSD scans were repeated at 6, 12, 24, and 48 hours later for a total of 5 observations per patient per eye in the ICU, thus resulting in a total of 400 ONSD measurements. The latter were performed by a single expert operator who was blinded to the patient's identity and to invasive ICP findings with the intention of minimizing bias. A Camino intraparenchymal catheter (Camino Laboratories, San Diego, CA, USA) was inserted by neurosurgeons in the frontal region of each patient. ICP measurements were continuously monitored and recorded simultaneously to ONSD measurements. Elevated ICP was defined as an ICP of 20 mmHg or greater [23].

### 2.3. Statistical Analysis

Summary data are expressed as mean ± standard deviation. Paired *t*-tests were used to compare the ONSD values between the left and right eyes of each patient. Initially, ONSD was correlated to ICP to establish linearity in a simple regression model. Subsequently, the impact of age, gender, weight, height, and ICP on ONSD was explored through a multiple regression nested (hierarchal) model. A multiple regression nested model simply means one model is a subset of another, where the independent variables are entered in the model at different levels, age and gender being the first level, followed by height and weight in the second, and ICP last. This model is known to produce unbiased estimates of the standard errors associated with the regression coefficients, and its goodness of fit is judged by the *r*^2^ change as nested models are sequentially entered [24]. Additionally, receiver operating characteristic (ROC) curves were obtained to specify cutoff values of ONSD and ICP. Cutoff values were the threshold values that maximized the sum of specificity and sensitivity. A two-tailed significance level of 0.05 was regarded statistically significant. All data were stored on a spreadsheet (Excel 2011; Microsoft, Seattle, WA, USA), and analyses were performed using a commercially available statistical package (SPSS version 24; IBM Corporation, Armonk, NY, USA).

## 3. Results

Table 2 presents the baseline features of the study population. A total of 40 patients with severe TBI were analyzed in this study. All patients underwent a baseline clinical evaluation and brain CT scan assessment. Patients exhibited a mean GCS of 4.5 ± 2.9 and a mean brain CT Marshall Scale of 4 ± 1.5 upon ICU admission. Concomitant injuries recorded upon admission included abdominal trauma (20%), thoracic trauma (40%), and other orthopedic injuries (65%). Thirty-four out of the 40 cases (85%) underwent various neurosurgical interventions (i.e., craniotomies, evacuation of subdural hematomas, and so on). In this cohort, males suffering from severe TBI were more than females and exhibited significantly higher ICP values (27.4, 95% CI 26.1 to 28.7 mmHg) compared to the latter (24.5, 95% CI 23.0 to 26.0 mmHg).

During the first 48 hours after admission, all patients underwent ONSD measurements as well as invasive ICP monitoring per study protocol as described previously. Both eyes were scanned for each patient, and the mean of both readings was correlated to simultaneous ICP recordings. One hundred seventy-seven (88%) of the 200 ONSD measurements for each eye were performed on patients with elevated ICP (>20 mmHg) who were managed according to the TBI protocol of our NCCU, while the remaining 23 (12%) were performed on patients with normal ICP recordings. The difference, although existent, in mean ONSD size between the left and right eyes was not significant (*p*=0.35; 6.9 mm (95% CI 6.7 to 7.0 mm) versus 6.8 mm (95% CI 6.7 to 7.0 mm), respectively).

**Figure 2:**
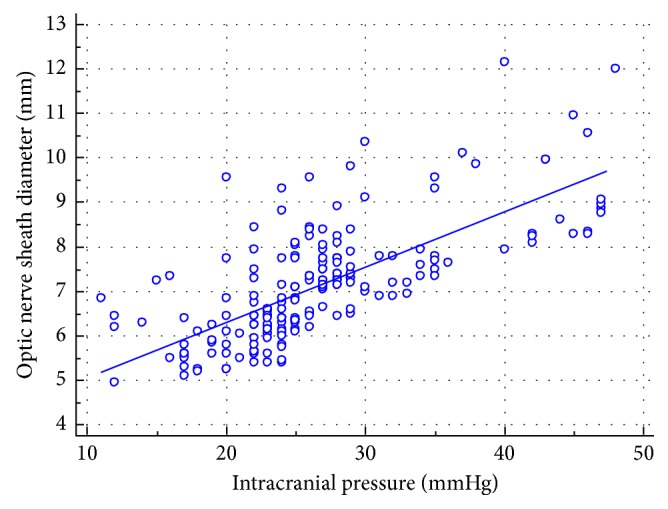
Mean optic nerve sheath diameter (ONSD) measurements plotted versus the invasive intracranial pressure (ICP) in the patients with severe traumatic brain injury.

In simple linear regression model, ONSD measurements were strongly correlated to invasively monitored ICP values (*r*=0.74, *p* < 0.0001; Figure 2). In the multiple regression nested model, ICP was a strong significant predictor of ONSD (standardized coefficient beta = 0.72, *p* < 0.001). In the same model, sex and height were significant predictors of ONSD, with *p* values of 0.006 and 0.04, respectively, whereas age (*p*=0.32) and weight (*p*=0.28) were not significant predictors in the model. The model was well fitted with *r*^2^ change from 8% to 55% after the addition of all the nested models (Table 3).

**Figure 3:**
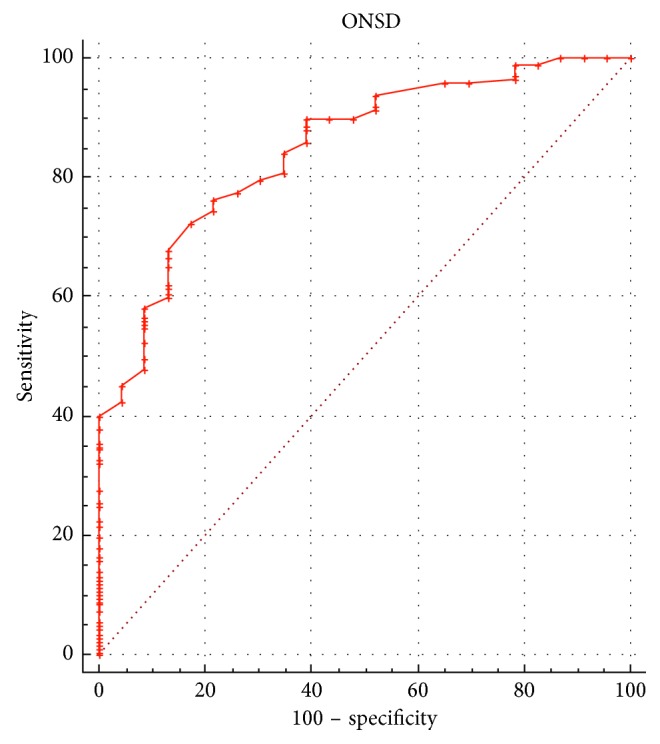
Receiver operating characteristic curve analysis showing the predictive value of the optic nerve sheath diameter (ONSD) measurements for elevated intracranial pressure (ICP; ≥20 mmHg). Diagonal segments are produced by ties.

**Figure 4:**
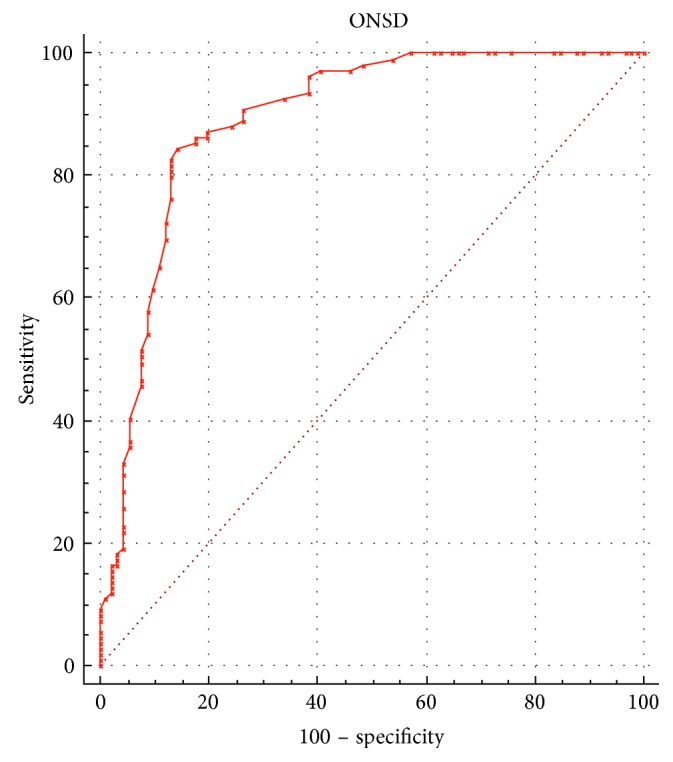
Receiver operating characteristic curve analysis showing the predictive value of the optic nerve sheath diameter (ONSD) measurements for elevated intracranial pressure (ICP; ≥25 mmHg). Diagonal segments are produced by ties.

The ROC curve analysis based on the diagnostic value of 20 mmHg or more for high ICP revealed that the optimal cutoff value of ONSD for predicting elevated ICP was 6.4 mm when using the mean of both eyes (area under the ROC curve = 0.88, 95% CI = 0.80 to 0.95; Figure 3). The sensitivity and the specificity of the cutoff value were 85.3% and 82.6%, respectively. Notwithstanding, when the ROC curve was repeated using a diagnostic value of 25 mmHg for high ICP, the optimal cutoff value of ONSD for predicting elevated ICP was 6.6 mm (AUC = 0.89, 95% CI = 0.84 to 0.94; Figure 4) baring a sensitivity of 87.2% and specificity of 80.2%, respectively, for the cutoff value.

In this cohort, five cases out of the 40 (12.5%) progressed towards cerebral circulatory arrest and were declared brain dead following pertinent clinical examination per hospital protocol. Four out of the 5 brain dead cases were harvested according to the regulations of the Saudi Center for Organ Transplantation (SCOT).

## 4. Discussion

Ultrasound evaluation of ONSD appears to be an increasingly popular noninvasive method of assessing ICP. Previous studies have shown a strong correlation between ICP and ONSD; however, there has been a substantial variability in the literature around the ideal ONSD cutoff value that corresponds to elevated ICP [7, 8, 10]. It has been proposed previously that one of the limitations around ONSD as a measure of ICP is the lack of standardization and quality control measures currently in place. In an effort to address this issue, newly published quality criteria for the sonographic assessment of ONSD were suggested (Table 1) [21]. In the present study, we applied the new quality criteria for the sonographic assessment of ONSD and compared the latter to invasive measurements of ICP by means of an intraparenchymal catheter.

In agreement with previous studies, we found a strong correlation between ONSD and ICP, and no significant difference between ONSD readings in the left and right eyes although differences were existent and documented. Interestingly, the value we calculated for the optimal cutoff point to determine elevated ICP from sonographic ONSD measurement (6.4 mm) is higher than most previously published values. In our ROC analyses, the area under the curve is quite large at 0.88, indicating high accuracy, which is similar to previous studies [8]. When a higher cutoff value (25 mmHg) for increased ICP was used in ROC analyses, the corresponding cutoff value for ONSD predicting high ICP was also found to be higher (6.6 mm), resulting in higher sensitivity but lower specificity nevertheless (Figures 3 and 4).

In the nested regression analysis, not surprisingly, most of changes in ONSD can be predicted by simultaneous changes in ICP. Also, we found a small but significant association between ONSD, sex, and height. The relationship of ONSD to height is interesting as it has not been previously observed in the literature [12–16]. This would require further validation in a larger cohort; moreover, our study is ongoing and, therefore, we would be able to validate the correlations in the future by analyzing a larger patient sample.

The difference in ONSD between genders in our study is consistent with data recently published in a cohort of healthy men and women [16]. However, such difference is higher in our study likely due to the features of our cohort who consisted entirely of patients with severe TBI. In severe TBI, ICP is more likely to be elevated, which could potentially augment the already naturally occurring difference in size between men and women's ONSD. Men are also more likely to suffer from a TBI, are more likely to be seriously injured, and have a grave prognosis [25] which may partially account for the significantly larger ONSD in these patients. In our study, males suffering from severe TBI were higher than females and exhibited significantly higher ICP values (27.4, 95% CI 26.1 to 28.7 mmHg) compared to the latter (24.5, 95% CI 23.0 to 26.0 mmHg). Further studies are required to analyze the effect of gender on ONSD measurements.

The present data suggest a reevaluation of previously defined thresholds for elevated ICP, either through new large prospective studies or remeasuring archived ONSD imaging utilizing the new criteria. These findings are important, as they indicate that previously published cutoff values for elevated ONSD generated using the black stripe method may not be applicable to images obtained using the new quality criteria. These higher cutoffs are similar to those seen when looking at ONSD as a predictor of elevated ICP in MRI studies [26].

The new quality criteria offer potential benefits such as standardization of ONSD measurements. The latter could translate to more accurate measurements between different sonographers, patients, or in the same patient over time. Hence, adopting new quality criteria opens the exciting possibility of monitoring ICP trend over time by ONSD measurements, a practice that has so far had disappointing results in the literature [22]. Notably, a large study showed that management based on measurement of ICP by intracranial catheter had no additional benefit compared to management based on clinical and imaging findings alone [27]. Despite lack of evidence of benefit, inaccessibility except in specialized centers, and risks of bleeding and infection, intracranial ICP monitoring is still the standard of care in patients with severe TBI [4]. More study is needed to determine whether ONSD can be safely used to diagnose ICP without the need of invasive monitoring or it will remain a noninvasive adjunct to help triage patients for intracranial catheter placement.

Despite the importance of our findings, there are several limitations to our study which could be mainly attributed to its inherent design and the small cohort of patients studied. Moreover, the results are likely only generalizable to the currently studied population. It has been previously shown that different ONSD cutoffs are useful for nontrauma and traumatic elevated ICP patients using the “black stripe” method [10], and we have no reason to believe this is different using the new quality criteria. Factors such as the acuity of elevation of ICP, comorbid medical illness, and co-occurring orbitofacial trauma all likely affect ONSD [21]. Also, pretest probability of raised ICP in the patient population affects the optimal ONSD cutoff to use [8]. Despite the rigorous quality criteria followed, ONSD is still an operator-dependent task that requires precise measurement of a 3–6 mm structure to the nearest 0.1 mm. Inherence in a measurement technique of this type will be erroneous due to intraobserver and interobserver variation, which has been studied elsewhere [20, 28–30] as in the current report we utilized a single operator to minimize bias. We found that ONSD measurements made using the new quality criteria could provide useful information for detecting elevated ICP in severe TBI. More study is clearly required to evaluate the new criteria for ONSD measurements in other patient populations as well as to determine whether ONSD might be used for ICP trend monitoring in brain-injured patients. Hopefully, this ongoing study might be able to answer some of the aforementioned raised points in the upcoming years.

## 5. Conclusion

This is the first study applying new quality criteria for the sonographic evaluation of ONSD. When applying the new quality criteria in TBI patients, ONSD measurements are highly correlated to invasive ICP values; moreover, a larger cutoff value of ONSD is evident as compared to past data (“black stripe” method). Also, a previously undetected correlation between ONSD and anthropometric data may exist. ONSD measurements may be used to help identify which TBI patients need ICP-lowering treatment, particularly when intracranial monitoring is contraindicated or not available. Further study is needed to determine how these new criteria affect ONSD measurement in other patient populations, and whether they can be used in the future to monitor ONSD as a proxy for ICP trend in brain-injured ICU patients.

## Figures and Tables

**Table 1:** Proposed sonographic quality criteria for optic nerve sheath diameter (ONSD) measurements (adapted from Sargsyan et al. [21]).

Sonographic quality criteria for optimization of ONSD measurements
(i) ONSD measurement should not be made through the lens (even the edge of the lens may not be visible on the image).
(ii) Sonographic differentiation (contrast) between the nerve proper and the arachnoid (cerebrospinal fluid space) must be obvious; measuring a “dark stripe” behind the globe without nerve and arachnoid differentiation is not acceptable.
(iii) The outer border of the arachnoid must be identifiable for actual ONSD measurement; clear, well-focused images must thus allow confident measurement of the inner diameter of the dural sheath.
(iv) Ideal views of the optic nerve demonstrate the point of its penetration into the globe, that is, “dark meets dark” (nerve meets vitreous without interposition of thick echogenic layer of the posterior sclera).
(v) Good views offer opportunities for additional information potentially useful with growing experience, such as tortuosity of the nerve, hypoechogenicity of the arachnoid, and its irregularity; this also allows seeing the optic disk area protrusion into the globe and flattening of the posterior globe in chronic ICP elevations (premorbid) that may mimic acute states in ICU.
(vi) Correct standardized measurements: since the most distensible portion of the sheath is at the 3-4 mm distance from the vitreoretinal interface, measurements are performed at this level in a direction perpendicular to the axis of the nerve.
(vii) It is highly recommended to measure ONSD bilaterally and in more than one image frame. This is an important quality assurance mechanism.
(viii) For ONSD trend monitoring, the previous record with images must be reviewed to ensure similar views and measurement technique. Prior images should be available at bedside (from the machine or in printout) for reference. ONSD measured in sagittal planes should not be compared with ONSD from axial planes.

**Table 2:** Baseline features of the study population upon admission.

Number of patients with severe TBI	*N*=40
Gender (male/female; %)	29/11 (73/27)
Age (years)	37 ± 16
Height (cm)	170 ± 10
Weight (kg)	72 ± 12
BMI (kg/m^2^)	24.9 ± 3.9
SBP (mmHg)	121 ± 18
DBP (mmHg)	69 ± 14
GCS (3–15)	4.5 ± 2.9
Marshall Scale (1–6)	4 ± 1.5
APACHE II score	21 ± 3.1
Concomitant injuries upon admission	
Orthopedic trauma (%)	26 (65%)
Chest trauma (%)	16 (40%)
Abdominal trauma (%)	8 (20%)

TBI: traumatic brain injury; BMI: body mass index; SBP: systolic blood pressure; DBP: diastolic blood pressure; GCS: Glasgow Coma Scale; APACHE II score: Acute Physiology and Chronic Health Evaluation II score.

**Table 3:** Multiple linear regression nested model for ONSD predictors.

Factor	Standardized *β*	*p* value
ICP	0.72	<0.001^∗^
Age	−0.07	0.32
Sex	−0.36	0.006^∗^
Height	0.10	0.04^∗^
Weight	−0.04	0.28

^∗^Statistical significance *p* < 0.05. ONSD: optic nerve sheath diameter.
